# Calibration‐free beam hardening correction for myocardial perfusion imaging using CT

**DOI:** 10.1002/mp.13402

**Published:** 2019-03-07

**Authors:** Jacob Levi, Brendan L. Eck, Rachid Fahmi, Hao Wu, Mani Vembar, Amar Dhanantwari, Anas Fares, Hiram G. Bezerra, David L. Wilson

**Affiliations:** ^1^ Department of Physics Case Western Reserve University Cleveland OH 44106 USA; ^2^ Department of Biomedical Engineering Case Western Reserve University Cleveland OH 44106 USA; ^3^ Research and Clinical Collaborations Siemens Healthineers Knoxville TN USA; ^4^ Philips Healthcare Cleveland OH 44143 USA; ^5^ Cardiovascular Imaging Core Laboratory Harrington Heart & Vascular Institute University Hospitals Case Medical Center Cleveland OH 44106 USA; ^6^ Department of Radiology Case Western Reserve University Cleveland OH 44106 USA

**Keywords:** beam hardening correction, cardiovascular imaging, CT, MPI‐CT, myocardial perfusion

## Abstract

**Purpose:**

Computed tomography myocardial perfusion imaging (CT‐MPI) and coronary CTA have the potential to make CT an ideal noninvasive imaging gatekeeper exam for invasive coronary angiography. However, beam hardening (BH) artifacts prevent accurate blood flow calculation in CT‐MPI. BH correction methods require either energy‐sensitive CT, not widely available, or typically, a calibration‐based method in conventional CT. We propose a calibration‐free, automatic BH correction (ABHC) method suitable for CT‐MPI and evaluate its ability to reduce BH artifacts in single “static‐perfusion” images and to create accurate myocardial blood flow (MBF) in dynamic CT‐MPI.

**Methods:**

In the algorithm, we used input CT DICOM images and iteratively optimized parameters in a polynomial BH correction until a BH‐sensitive cost function was minimized on output images. An input image was segmented into a soft tissue image and a highly attenuating material (HAM) image containing bones and regions of high iodine concentrations, using mean HU and temporal enhancement properties. We forward projected HAM, corrected projection values according to a polynomial correction, and reconstructed a correction image to obtain the current iteration's BH corrected image. The cost function was sensitive to BH streak artifacts and cupping. We evaluated the algorithm on simulated CT and physical phantom images, and on preclinical porcine with optional coronary obstruction and clinical CT‐MPI data. Assessments included measures of BH artifact in single images as well as MBF estimates. We obtained CT images on a prototype spectral detector CT (SDCT, Philips Healthcare) scanner that provided both conventional and virtual keV images, allowing us to quantitatively compare corrected CT images to virtual keV images. To stress test the method, we evaluated results on images from a different scanner (iCT, Philips Healthcare) and different kVp values.

**Results:**

In a CT‐simulated digital phantom consisting of water with iodine cylinder insets, BH streak artifacts between simulated iodine inserts were reduced from 13 ± 2 to 0 ± 1 HU. In a similar physical phantom having higher iodine concentrations, BH streak artifacts were reduced from 48 ± 6 to 1 ± 5 HU and cupping was reduced by 86%, from 248 to 23 HU. In preclinical CT‐MPI images without coronary obstruction, BH artifact was reduced from 24 ± 6 HU to less than 5 ± 4 HU at peak enhancement. Standard deviation across different regions of interest (ROI) along the myocardium was reduced from 13.26 to 6.86 HU for ABHC, comparing favorably to measurements in the corresponding virtual keV image. Corrections greatly reduced variations in preclinical MBF maps as obtained in normal animals without obstruction (FFR = 1). Coefficients of variations were 22% (conventional CT), 9% (ABHC), and 5% (virtual keV). Moreover, variations in flow tended to be localized after ABHC, giving result which would not be confused with a flow deficit in a coronary vessel territory.

**Conclusion:**

The automated algorithm can be used to reduce BH artifact in conventional CT and improve CT‐MPI accuracy particularly by removing regions of reduced estimated flow which might be misinterpreted as flow deficits.

## 1 Introduction

**Figure 1:**
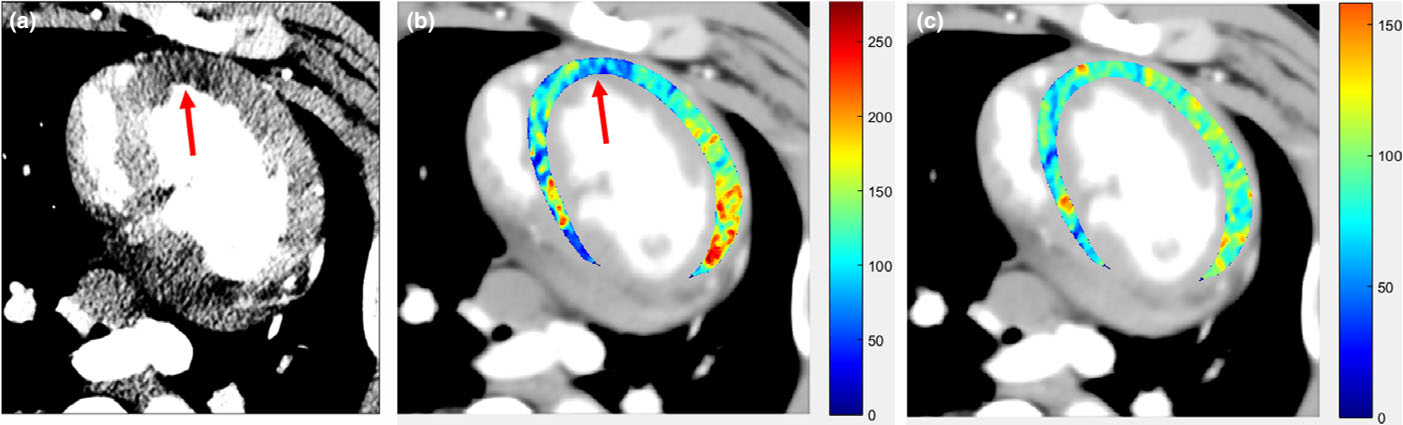
Beam hardening artifact and its influence on blood flow estimation in a normal pig heart without coronary obstruction. (a) BH artifacts in conventional CT images appear as dark streaks between high‐attenuating structures. (b) Blood flow map calculated from the conventional 120kVp images. A false blood flow deficit can be seen (arrow) at a much lower flow than at about 4 o'clock. (c) Blood flow map calculated from the spectral detector CT (SDCT) 70 keV images. Blood flow is much more homogeneous as SDCT significantly reduces BH artifacts. [Color figure can be viewed at wileyonlinelibrary.com]

Myocardial perfusion imaging using computed tomography (MPI‐CT) and coronary CTA have the potential to be noninvasive gatekeepers for coronary angiography. However, beam hardening (BH) artifacts hinder reliable interpretation of MPI‐CT images as well as accurate blood flow measurement from dynamic perfusion. BH is of particular importance in MPI‐CT because of the large amount of iodine in the heart ventricles and aorta. In CT reconstructions, BH results in dark streaks evident in the myocardium, which can be in the same order of magnitude as myocardium enhancement. A dynamic MPI‐CT scan is usually comprised of a set of 30–40 volumes containing different iodine concentrations. Accurately correcting each one of those volumes is essential for calculating accurate blood flow, making MPI‐CT one of the most demanding applications for BH correction. Often, BH is difficult to fully interpret visually, leading to false‐positive readings of individual CT images^1^ and often result in inaccurate quantification of blood flow from dynamic sequences. Quantitatively, reductions in HU values in the myocardium may lead to underestimation of myocardial blood flow (MBF) from dynamic CT‐MPI data as shown in Fig. 1.

There are many BH reduction and correction approaches. One physical approach is to use prefiltration to harden the x‐ray beam and therefore reduce BH in the final reconstruction.^2^, ^3^ Prefiltration is achieved using a filter comprised of different layers of materials (e.g., aluminum and copper) and a bow‐tie filter. With energy‐sensitive CT scanners (e.g., kVp switching, spectral detector, or dual source), one can create virtual monoenergetic images that are relatively free from BH artifacts. Another approach uses a phantom with known characteristics to measure a scanner‐specific material calibration curves and correct projections before reconstruction.^2^, ^3^, ^4^, ^5^ This type of method is used in most CT scanners to achieve water BH correction. There have been many BH correction solutions proposed that use image content, including the one presented in this paper.^2^, ^3^, ^4^, ^5^, ^6^, ^7^, ^8^, ^9^ In general, a preliminary image is reconstructed and used to obtain an estimate of material distribution and measurement error due to BH artifacts. The post‐reconstruction algorithms can be divided into two groups: projection‐based and image‐based corrections. Projection‐based algorithms take advantage of the raw data and sometimes other scanner characteristics such as x‐ray tube spectrum, source‐to‐detector geometry, and periodically obtained calibration tables. While detailed knowledge of the CT system leads to suitable BH correction, this requires scanner‐specific knowledge and raw data. One can correct for BH artifact using knowledge of the x‐ray spectrum^10^ or, in the absence of a known spectrum, using Poisson noise to estimate material attenuation properties^11^ or using projection consistency.^12^, ^13^, ^14^, ^15^ However, when raw data are unavailable, as in many cases, image‐based approaches are needed. Image‐based approaches are mainly based on segmentation of the input image into low‐ and high‐attenuating materials then forward projecting the segmented images to estimate the original sinogram and relative contribution from different materials.^4^, ^5^ Once forward projections are obtained, BH corrections can be performed. Iterative algorithms^2^, ^5^, ^9^, ^10^, ^16^, ^17^, ^18^, ^19^, ^20^, ^21^, ^22^, ^23^, ^24^, ^25^, ^26^, ^27^, ^28^, ^29^, ^30^, ^31^, ^32^ are very popular and usually work by iteratively improving an image using one of the previously described methods until some stopping criterion is met. Some of those algorithms are applied as part of the iterative reconstruction process used in some scanners and apply additional projection corrections like in the case of the iterative BHC (IBHC)^10^ or the dynamic IBHC (DIBHC).^20^ Usually in those cases, a cost function will measure the “distance” between the next iteration's image to an original image, usually obtained using filtered back projection (FBP), and add cost to another measured quantity like image noise. By iteratively improving the image, the final reconstructed image can achieve a significant noise reduction. A different kind of iterative BHC works by iteratively optimizing correction parameters until a cost function is minimized, for example, the method presented in this work. Most post‐reconstruction algorithms require prior knowledge of different scanner parameters, like the x‐ray tube spectrum, and/or require calibration for a specific scanner. In our work, we aim for a scanner‐independent, calibration free, image‐based solution requiring no access to raw data but only reconstructed images.

To reduce the need for calibrations and to enable perfusion assessments on any scanner, we propose an image‐based, calibration‐free, automatic beam hardening correction (ABHC) algorithm. The method iteratively optimizes correction parameters to minimize a BHA‐sensitive cost function. We quantitatively evaluate ABHC by applying it to a variety of digital and physical phantoms, as well as to preclinical and clinical images. Because we use an SDCT scanner, we can elegantly compare in detail conventional CT, conventional CT with ABHC, and virtual keV (without BH) acquired in the same acquisition.

### 1.A. Theory

The proposed ABHC method uses an iterative approach to estimate polynomial correction parameters. We first describe the polynomial BH correction method. We then describe the ABHC algorithm and processing pipeline.

### 1.B. Polynomial beam hardening correction

We derive the polynomial beam hardening correction, a method described in more detail elsewhere.^33^ The intensity of a monoenergetic x‐ray beam passing through a homogeneous material with linear attenuation coefficient μ can be calculated using Beer's law:

(1) $$I=I_{0}e^{-\int \mu dx}$$

where *I*_0_ is the initial beam intensity. The projection, represented in a sinogram, is obtained by:

(2) $$P=\ln\left(\frac{I_{0}}{I}\right)=\int \mu dx$$

From Eq. (2), one can see that the relationship between the material thickness and the projection P is linear for a homogeneous material. For a heterogeneous material, Eq. (2) would be modified by replacing μ with (*x*).

In the case of a polychromatic x‐ray beam passing through a material with energy dependent linear attenuation coefficient μ(*E*), Eq. (1) takes the form of Eq. (3), where Ω(*E*) is the normalized spectrum of the x‐ray beam.

(3) $$I=I_{0}\int \Omega \left(E\right)e^{-\int \mu (E)dx}dE$$

**Figure 2:**
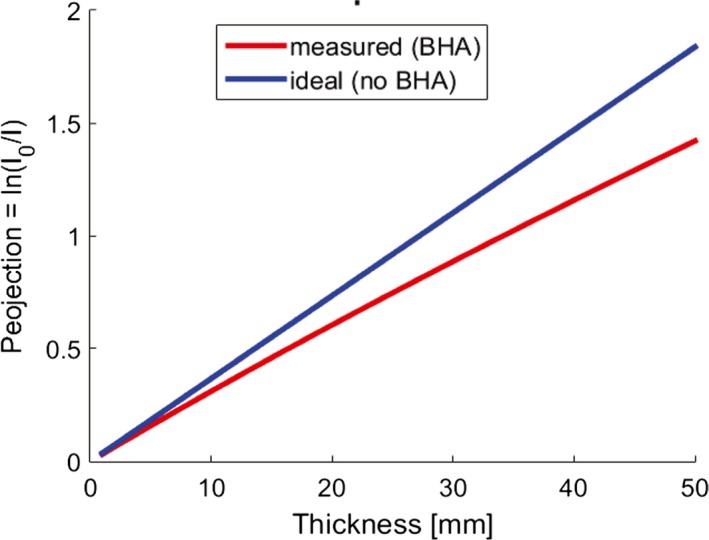
Beam hardening causes underestimation of the linear attenuation as given by $\ln\left(I_{0}/I\right)$. Data were simulated using monoenergetic source (ideal), and a polyenergetic source (realistic) passing through Cortical bone. Cortical bone μ(*E*) values were taken from NIST.^42^ [Color figure can be viewed at wileyonlinelibrary.com]

In this case, the projection, given by Eq. (4), is a nonlinear function of the material thickness (due to beam hardening). In this case, the projection is underestimated as compared to the monoenergetic case (Fig. 2).

(4) $$P=\ln\left(\frac{I}{I_{0}}\right)=\ln\left(\int \Omega \left(E\right)e^{-\int \mu \left(E\right)dx}dE\right)$$

In order to correct BH, we map the measured projection P (illustrated by the red line in Fig. 2) to a corrected line P_c_ (illustrated by the blue line in Fig. 2) using a polynomial expansion, as presented in Eq. (5), where N denotes the order of the polynomial. This method is applied in current CT scanners for water correction.^2^, ^3^, ^4^, ^5^ In this case, the coefficients *α*_*i*_ can be calculated from the knowledge of water's linear attenuation coefficients and their dependency on energy, or in an empirical way using water phantoms.

(5) $$P_{c}\approx \sum_{i=1}^{N}\alpha_{i}P^{i}$$

In contrast‐enhanced cardiac imaging, such as MPI‐CT, where there are additional highly attenuating material (HAM) objects, like bone and iodine, water correction is insufficient. Since the projection is a sum of different μ′*s* along the x‐ray beam [Eq. (2)], we can commutate the addends and, without loss of generality, treat the scanned object as a combination of one slab of soft tissue, or water‐equivalent, and one slab of HAM. The same argument applies to the polyenergetic case [Eq. (4)], since we treat each energy separately and then integrate over them. In this case Eqs. (4) and (5) can be re‐written as

(6) $$P_{c}\approx \sum_{i=1}^{N}\alpha_{i}ln^{i}\left(\frac{I_{0}}{I_{w}}\right)+\sum_{i=1}^{N}\beta_{i}ln^{i}\left(\frac{I_{w}}{I}\right)$$

where *I*_*w*_ denotes the beam's intensity after passing through the water (soft tissue) component.

Water‐based BH correction is automatically performed on most commercial CT scanners. To find the residual BH, *ΔP*, due to HAMs, we can subtract Eq. (6) from Eq. (5) to form:

(7) $$\Delta P\approx \sum_{i=1}^{N}\alpha_{i}ln^{i}\left(\frac{I_{0}}{I}\right)-\left[\sum_{i=1}^{N}\alpha_{i}ln^{i}\left(\frac{I_{0}}{I_{w}}\right)+\sum_{i=1}^{N}\beta_{i}ln^{i}\left(\frac{I_{w}}{I}\right)\right]\approx \sum_{i=1}^{N}\left(\alpha_{i}-\beta_{i}\right)ln^{i}\left(\frac{I_{w}}{I}\right)=\left(\alpha_{1}-\beta_{1}\right)\lambda +\left(\alpha_{2}-\beta_{2}\right)\lambda^{2}+\ldots =a\lambda +b\lambda^{2}+\ldots$$

where *λ* $\overset{\mathrm{def}}{=}$ln(*I*_*w*_/*I*), and can be found by thresholding the original image to obtain the HAM image and then forward projecting it. In our work, we are using the second order polynomial, as have others.^34^ The error image *I*_*E*_ is then generated as the FBP of *ΔP*. The final BH corrected image is obtained by:

(8) $$I_{C}=I-I_{E}$$

Since λ is the projection only through HAM, *aλ* can only change the intensity and cupping of the HAM, due to the linearity of the FBP reconstruction, but cannot reduce streak artifacts due to BH. The quadratic term *λ,* 2 on the other hand, is responsible for reducing streak BH artifacts.

### 1.C. Automatic beam hardening correction

**Figure 3:**
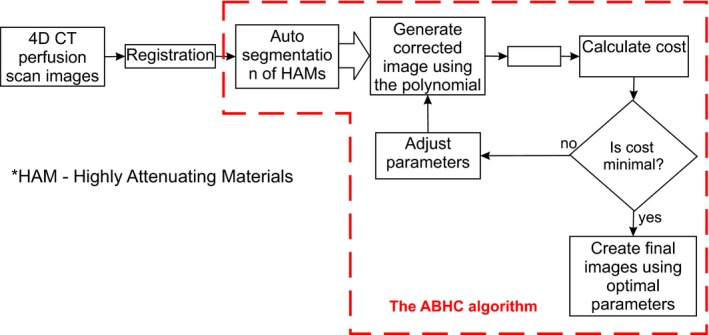
Flow chart of the ABHC algorithm. [Color figure can be viewed at wileyonlinelibrary.com]

The ABHC iteratively optimizes a BHA‐sensitive cost function to estimate polynomial parameters to create BH‐corrected images.^1^, ^6^ As shown in Fig. 3, ABHC begins by segmenting the HAM by using a threshold. ABHC will then apply the chosen BH correction algorithm to an input image using initial parameters. The corrected image is then filtered, and a BH‐specific cost function is evaluated. The algorithm iteratively adjusts the BH correction algorithm parameters until the cost is minimized. The final corrected image is generated using the optimized parameters.

To apply ABHC to dynamic perfusion images, we first preprocess the images. We register the cardiac volumes obtained over time using a nonrigid registration method described previously for ECG‐gated image acquisitions.^35^ Each volume is registered to the peak enhanced volume and ABHC correction parameters are determined and applied in one of the following ways. First, parameters can be estimated using a single image volume at peak enhancement and then use those parameters to correct all other images (ABHC‐peak). Second, parameters can be estimated for each image containing iodine in the stack, and then apply the average of those parameters to the entire dataset (ABHC‐average). Third, parameters can be estimated and applied for each image separately (ABHC‐single).

Segmentation of the left ventricle (LV) cavity and myocardium, needed for calculation of the cost function, is performed as follows. First, we analyze the registered time series to identify constant, high HU structures (bones), and structures that change intensity over time (iodine enhanced). We obtain the bone and iodine masks by thresholding the images. We then compute the standard deviation (SD) of each pixel over time. Structures that are not enhanced with iodine will have an SD equivalent to the image noise. Structures that are slightly enhanced, like the myocardium, will have a higher SD. Structures with the highest SD are cavities that are filled with iodine, like the LV and aorta. Out of all the slices in the volume and over time, the slice with the largest sum of all temporally enhanced pixels will be the slice that presents the peak enhanced time point. On that slice, we apply connected components to all structures having an SD above a threshold. The largest structure will be the LV. Using connected components, we find the LV in other slices. To segment the myocardium, we dilate the LV mask and subtract the original LV mask to get a 3D shell mask containing the myocardium. The LV mask dilation parameters are chosen in a way that the dilated mask will contain the whole myocardium. We observed several hearts in a partial field of view (FOV), in which the myocardium looks the biggest, and found that dilation with a disk where r = 20 covers all cases. We then mark only those pixels from the shell mask with an SD appropriate for the myocardium to obtain the segmented myocardium. The SD parameter is determined by classifying it to three classes: static structures in which SD comes from the noise of the image; blood pools, like the LV or aorta, which will have a large SD due to the quick perfusion; and the myocardium, that has an SD in between. Disregarding noise, the SD values are ~0, ~20, and well above 100 for static, myocardium, and blood pools respectively.

Noise reduction filtering is an important preprocessing step prior to computing our cost function. High‐frequency noise, particularly textured streaks in reconstructed images, interferes with assessment of low‐frequency BH artifacts in the cost function. We experimented with different simple noise‐reduction filters (Gaussian, median, and combinations) and determined the effect on BH corrections.

### 1.D. Creating a corrected image

The algorithm starts by segmenting HAM and forward projecting it to find *λ* [Eq. (7)]. Then, *λ* ^2^ is calculated and FBP to obtain ${\hat{I}}_{\mathrm{HAM}}^{2}$. Next, the error image *I*_*E*_ is constructed: $I_{E}=a\cdot I_{\mathrm{HAM}}+b\cdot {\hat{I}}_{\mathrm{HAM}}^{2}$. The base images *I*_*HAM*_ and ${\hat{I}}_{\mathrm{HAM}}^{2}$ are found only once which significantly reduces optimization time. Finally, the corrected image *I*_*C*_ is obtained by: *I*_*C*_ = *I* − *I*_*E*_. The iterative process finds “a” and “b” that minimizes the cost function that is calculated over the corrected image *I*_*C*_.

### 1.E. Cost function

We created a cost function sensitive to BH artifacts that typically manifest as dark and bright regions in the image, as dark regions between two HAMs, and as cupping artifacts within homogeneous HAM regions. The cost function consists of two terms, where the first term, *TV*_*myo*_, addresses bright and dark artifacts in the myocardium and the second, *F*_*LV*_, addresses cupping artifacts in the LV. The cost function is given by:

(9) $$\psi \left(I_{C}\left(x,y|\theta \right)\right)=\alpha \cdot TV_{\mathrm{myo}}\left(\hat{f}\left(I_{C}\left(x,y|\theta \right)\right)\right)+\left(1-\alpha \right)\cdot F_{\mathrm{LV}}\left(I_{C-LV}\left(x,y|\theta \right)\right)$$

where *Ψ* is the cost of the corrected image, *I*_*C*_(*x*,* y*|*θ*), generated using the BH correction algorithm parameter vector *θ*. The first and second terms in Eq. (9), assess streak artifacts and cupping, respectively. The coefficient α determines the relative weight of each term in the cost function. The first term is a measure of the total variation (TV) within the myocardium, TV_myo_. Since BHA is a relatively low‐frequency artifact compared to noise, the image is first filtered, using a Gaussian filter $\hat{f}$. That ensures that the BH contribution to the TV term will not be overwhelmed by the TV of noise. TV_myo_ is then computed using the following TV formula:

(10) $$\begin{array}{c} G_{x}=\frac{\partial \hat{f}\left(I_{C}\left(x,y|\theta \right)\right)}{\partial x},G_{y}=\frac{\partial \hat{f}\left(I_{C}\left(x,y|\theta \right)\right)}{\partial y},G^{\prime }=G_{x}^{2}+G_{y}^{2}\\ TV_{\mathrm{myo}}\left(\hat{f}\left(I_{C}\left(x,y|\theta \right)\right)\right)=\sqrt{\sum_{\mathrm{myo}}G^{\prime }}/A_{\mathrm{myo}} \end{array}$$

G′ is the gradient magnitude of G, and is obtained by thresholding G such that all gradient values above a threshold are set to zero. This step is done in order to calculate only the gradient on relatively flat signals within the myocardium and not on high‐magnitude edges like those between the myocardium and the LV, or the myocardium and the air in the lungs. *A*_*myo*_ is the area of the myocardium in pixels and is used to normalize *TV*_*myo*_. The second term, *F*_*LV*_, assesses cupping. Cupping is a well‐known phenomenon in CT images that manifests as artificial darkening toward the middle of homogeneous attenuating structures in the image. Using the segmented LV in the corrected image ($I_{C-LV}\left(x,y|\theta \right)$), we calculate the mean of the 20 highest HU values on the rim of the LV and subtract the average noise over the rim to obtain *LV*_*max*_. We subtract the average noise in order to eliminate the bias that is introduced by choosing the 20 highest HU values. The rim of the LV is four pixels wide, along the circumference of the LV. We then calculate the sum of square differences between every pixel in the LV to *LV*_*max*_:

(11) $$F_{\mathrm{LV}}\left(I_{C-LV}\left(x,y|\theta \right)\right)=\sqrt{\sum_{\mathrm{LV}}{\left(I_{C}\left(x,y|\theta \right)-LV_{\max}\right)}^{2}}/A_{\mathrm{LV}}$$

where *A*_*LV*_ is the area of the LV and is used to normalize the *F*_*LV*_ term.

## 2 Experimental methods

### 2.A. Blood flow estimation

We used a model‐based deconvolution algorithm to estimate MBF from dynamic, contrast‐enhanced CT images. Our previous work has suggested model‐based deconvolution to be more accurate than model‐independent deconvolution.^36^ After segmenting the myocardium (this segmentation determines where blood flow will be calculated to generate flow maps and it is not a part of ABHC) using a semi‐automated algorithm (Medis), we compute average myocardial time‐attenuation curves (TACs) over 5 × 5 super‐pixel regions within the myocardium. We also obtain an average arterial input function (AIF) from a circular region in the center the LV cavity as defined by the user. The AIF is convolved with an analytic impulse response function (IRF) like the one used in the Johnson‐Wilson model^35^, ^37^, ^38^ to produce a model tissue TAC. The IRF is defined by five parameters: time delay, flow, intravascular transit time, extraction fraction, and a decay constant. We reduce the model to three free parameters similar to other works.^39^ Specifically, we fix the intravascular transit time^40^ (ITT = 2 s) and extraction fraction (E = 0.6), which are within the expected physiologic range and provided good fits to physiologic data.^40^, ^41^ To reduce the effect of numerical errors in the model tissue TAC, convolution is performed at 100 ms time increments with the AIF linearly interpolated between measured time points. The sum of squared difference (SSD) is used as the cost function between the model TAC and the measured TAC at the sampled time points. The SSD is minimized as a function of the model parameters using a Nelder‐Mead simplex algorithm in Matlab (fminsearch).

In porcine experiments on an animal that does not suffer from cardiovascular disease, we anticipate homogeneous perfusion. To evaluate, we calculated flow ratios whereby we divided the estimated flow in a large ROI with the lowest flow by the estimated flow in an ROI with the highest flow.

### 2.B. Artifact measurements

We quantified BH artifact in images by multiple ways. (a) We measured the difference in average HU inside the BH artifact ROI to the average HU in a remote, unaffected ROI. If the BH affected ROI was darker than an unaffected ROI, we got a negative BH artifact value. (b) Cupping in what should be a homogenous region was measured as the difference between the mean HU value taken in a small ROI in the darkest point in the region and the mean HU of a small ROI in the brightest point in the region. Averaging over an ROI helps to reduce bias introduced by noise and other artifacts like Gibbs ringing. Percent cupping reduction was calculated as the absolute difference between the original cupping and the cupping as measured on the corrected image, divided by the original cupping. (c1) In order to measure homogeneity along the myocardium, we measured the mean HU values in several ROIs along the myocardium and calculated their standard deviation.

### 2.C. Digital and physical phantoms

**Figure 4:**
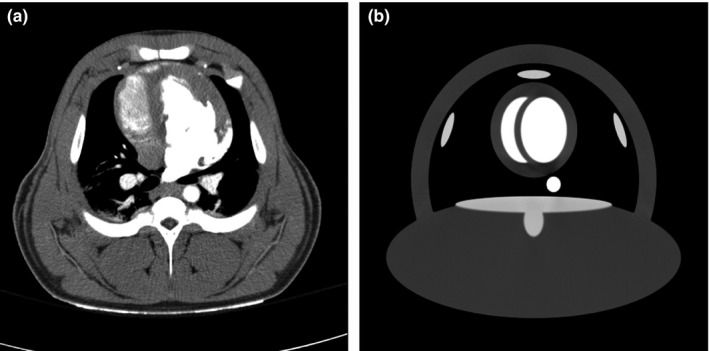
Simulated porcine phantom. (a) A porcine scanned on a prototype Philips SDCT scanner using a cardiac perfusion protocol. (b) Simulated cardiac porcine phantom. W = 60/L = 360.

Because of the ability to know ground truth, we evaluated ABHC using digital phantoms created within a CT simulator. The CT simulation software models a realistic CT scanner (Brilliance 64, Philips), with a cone beam source, finite width detector grid, x‐ray prefiltration, and x‐ray spectrum. Virtual objects are created with 3D geometric primitives (e.g., ellipsoidal volume). The simulation computes line integrals based on analytic object geometries with defined mass‐attenuation spectra from NIST^42^ and accounts for Poisson noise. We created two simulated phantoms: a water cylinder with four tubes filled with different concentrations of iodine (shown later in Fig. 6) and a cardiac porcine phantom (Fig. 4). We used the cardiac phantom to simulate both static images and dynamic perfusion scans. To generate an image sequence, we used a physiologic perfusion simulator^39^ to generate appropriate, homogeneous iodine attenuation curves for the heart chambers and myocardium. We simulated both polyenergetic (e.g., 120 kVp) and monoenergetic (70 keV) x‐ray sources. The reason we chose 70 keV as the optimal energy for these experiments is discussed in depth in our previous publication.^43^ Briefly, 70 keV HU values closely align with conventional reconstruction on this scanner and provide minimal BH artifacts while maintaining high CNR and SNR. The monoenergetic simulation gave us ground truth since there were no BH artifacts. We simulated a homogeneous MBF of 100 ml/min/100 g. Digital phantom MBF maps were evaluated both qualitatively and quantitatively against the known simulated value.

To account for all measurement issues, we constructed and imaged two physical phantoms. The first phantom had diameter of 23 cm and a thickness of 6 cm with four test tube insets filled with iodine concentrations of 15, 22, 26.5, and 77 mgI/ml. The second phantom was 6 cm thick with 26 cm diameter and eight test tube insets. This phantom was scanned with low‐concentration iodine insets (0, 0.875, 1.75, 3.5, 5.25, 7, 8.75, and 10.5 mgI/ml) and with high‐concentration insets (0, 1.75, 3.5, 7, 10.5, 14, 17.5, and 21 mgI/ml), hereafter called low‐ and high‐concentration phantoms, respectively.

### 2.D. Parameter optimization

The weighting parameter α, which sets the relative weights of the two parts of the cost function, was found empirically. Thirteen pigs and simulated phantoms were corrected using a grid search on α. The optimal α was found for each subject visually, that is, the α that produced an image with minimal BH artifacts. Finally, the value of α was calculated as the average of those individual α's.

### 2.E. CT imaging

We imaged phantoms and *in vivo* porcine using a prototype spectral detector CT (SDCT, Philips Healthcare). The SDCT is well‐suited to this study as it is capable of reconstructing virtual monoenergetic images which greatly reduce BH artifacts, as well as conventional CT images with BH artifacts, from the same scan. For each scanning experiment, we compared conventional CT, conventional CT with ABHC, and 70 keV images. In the case of *in vivo* perfusion experiments, we also compared perfusion measurements between different data sets. The dynamic MPI‐CT protocol included 40 ECG‐gated scans acquired at 45% R‐R cycle (near end systole), 120 kVp, 100 mAs, 4 cm coverage, full 360‐degree scans, and 0.27 s rotation speed. Static imaging was done with similar settings. Standard and virtual monoenergetic (70 keV) image slices of 512 × 512 pixels were reconstructed with 2 mm slice thickness, 2 mm increment, and 120 mm FOV. In some experiments, we acquired images on a conventional CT scanner, Brilliance iCT (Philips Healthcare) using similar protocols.

### 2.F. Preclinical *in vivo* imaging study

Our porcine model, as described elsewhere,^35^, ^43^ used Yorkshire female, weight: 40–50 kg, age: 13–15 weeks with percutaneously induced ischemia. A small angioplasty balloon was inserted in the left anterior descending (LAD) artery, guided by a fractional flow reserve (FFR) wire.^44^ This allowed us to accurately induce ischemia in LAD territory of the myocardium. The balloon was inflated to induce the desired level of flow restriction as determined by FFR. We used the FFR = 1.0 condition to evaluate the ability of ABHC to reduce BH artifact and give uniform perfusion while the FFR = 0.7 condition was used to evaluate whether ABHC preserves ischemia. All experiments were conducted under IACUC approval.

## 3 Results

### 3.A. Optimization of the algorithm

**Figure 5:**
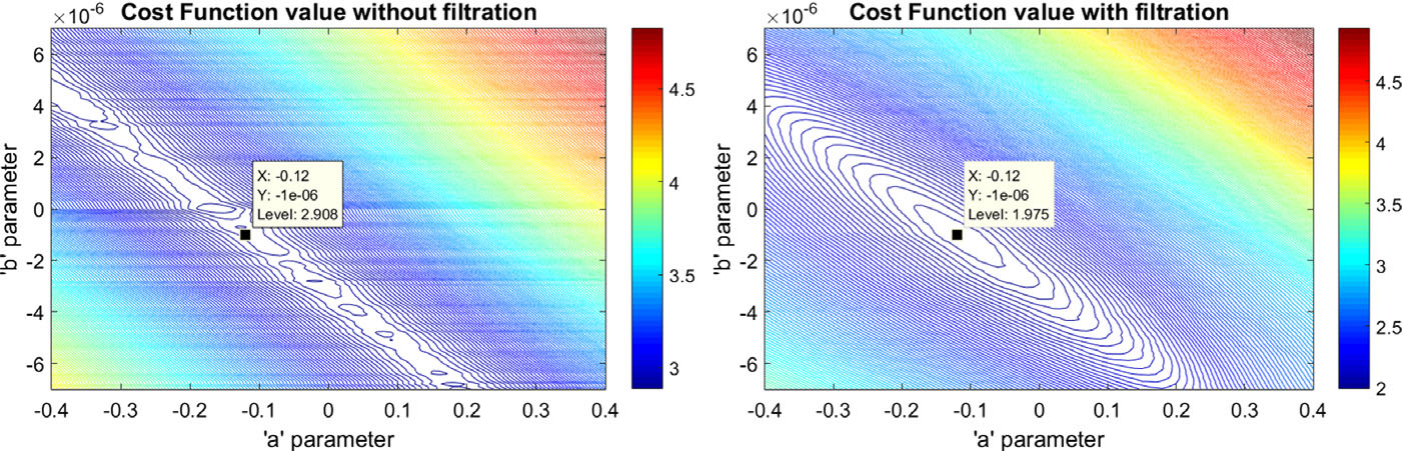
Cost function value as a function of correction parameters “a” and “b,” with and without filtration. The data cursor on both panels shows the optimal correction parameters. Left: without filtration there are several local minima and the global minimum is not the same as that with noise reduction. Right: after filtration, there is a clear global minimum. Visual examination of resulting images supported the global minimum after noise reduction. [Color figure can be viewed at wileyonlinelibrary.com]

From optimization experiments starting with different initializations, we discovered that it was necessary to reduce noise in the intermediate BH‐corrected images to avoid capture in local minima. Figure 5 shows that without filtration, the cost function has multiple local minima that can capture an optimization algorithm. However, after noise reduction, there is a smooth descent of the cost function to a single global minimum, at the same parameter values as obtained without noise reduction. We experimented with filters including median, median followed by linear and linear filtering, where linear filtering was done with Gaussian filters of different sizes. Best results were obtained with 2D Gaussian filtration (σ=0.7mm, ~3 pixels). With this filtration, optimization was robust as we could initialize at different parameter values and still converge to the same minimum.

The cost function is comprised of two parts (TV of the myocardium and cupping artifact) as shown in Eq. (9). In experiments, we found that both parts contributed to the accuracy and robustness of the ABHC algorithm. Although the TV term was enough to find the “b” parameter, we obtained poor accuracy for the “a” parameter without including the cupping term. That is, without the cupping term, the “a” parameter had large fluctuations between consecutive slices with average difference of ±0.098. By adding the cupping term, this variation was reduced to less than ±0.01. The coefficient *α*, which sets the relative importance of the two cost function terms was optimized in a grid search and found to be *α* = 0.47 ± 0.04 over 13 pig experiments and simulations. Furthermore, we found that the cupping term improved robustness in cases of mild ischemia. By setting *α* = 1, we were able to disable the flatness term of the cost function. In this case, we observed that TV was able to slightly over‐correct BH, leading to partial obscuring of mild ischemia (only where BH artifact was present). Adding the cupping term prevented this. We did not observe this in healthy heart (FFR = 1) and in fully occluded LAD (FFR <=0.3).

In order to apply BH correction to a set of dynamic MPI‐CT images, we tested multiple approaches. First, we found that “ABHC‐single” introduced undesirable fluctuations in corrected intensity values over time that impaired MBF estimates. In “ABHC‐average,” we averaged correction parameters found when there was significant iodine present in the image and applied them to all images in the set. ABHC‐average produced good results but was time‐consuming. In “ABHC‐peak,” we used parameters estimated at the peak of the contrast in the image. This gave us comparable results to ABHC‐average with a shorter execution time. In the best ABHC‐hybrid method, we identified the peak contrast image, estimated values at this image and the two adjoining images in time, estimated parameters for teach image, and averaged them. That gave us very good, robust results with a reasonable execution time. In order to estimate blood flow on the whole heart volume, that is, all spatial slices, we used the correction parameter obtained from one slice in the middle of the volume, where the area of the LV is the largest, and applied it to all other slices.

### 3.B. Experimental results

**Figure 6:**
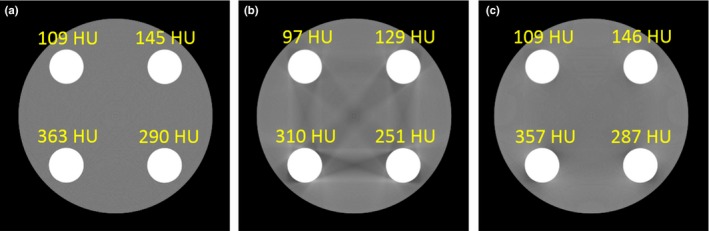
Reduction of BH artifacts using ABHC in a simulated phantom. (a) Original phantom simulated without noise in order to isolate BH artifacts. (b) Conventional FBP reconstruction. (c) ABHC corrected image. W = 80/L = 0. [Color figure can be viewed at wileyonlinelibrary.com]

**Figure 7:**
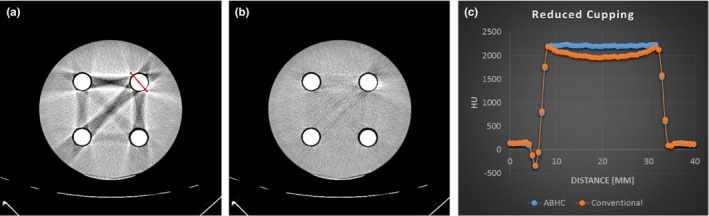
Reduction of BH artifacts using ABHC in physical phantom. (a) Acrylic phantom with four inserts filled with 15, 22, 26.5, and 77 mgI/ml (starting at bottom right and moving clock wise) reconstructed with FBP. (b) BH‐corrected image using ABHC. (c) Cupping artifact comparison. BH streak artifacts were reduced from 48 ± 6 HU to 1 ± 5 HU and cupping was reduced by 86%. W = 100/L = 100. [Color figure can be viewed at wileyonlinelibrary.com]

The ABHC algorithm was applied to static digital and physical phantoms (Figs. 6 and 7). With the digital phantom (Fig. 6), we found that streak artifacts are barely seen following ABHC with artifacts reduced from roughly 13 ± 2 HU to 0 ± 1 HU in the most affected region, near 3 o'clock. HU values following correction matched phantom values in iodine‐containing cylinders and background within ±2%. Results from the physical phantom show similar reduction in BH artifact (Fig. 7). The apparent increase in artifacts is due to the use of high concentrations of iodine to accentuate the artifact. BH artifact was reduced from 48 ± 6 HU to 1 ± 5 HU [Fig. 7(b)]. Cupping within iodine‐containing vials was reduced by 86%, from 248 to 23 HU [Fig. 7(c)].

**Figure 8:**
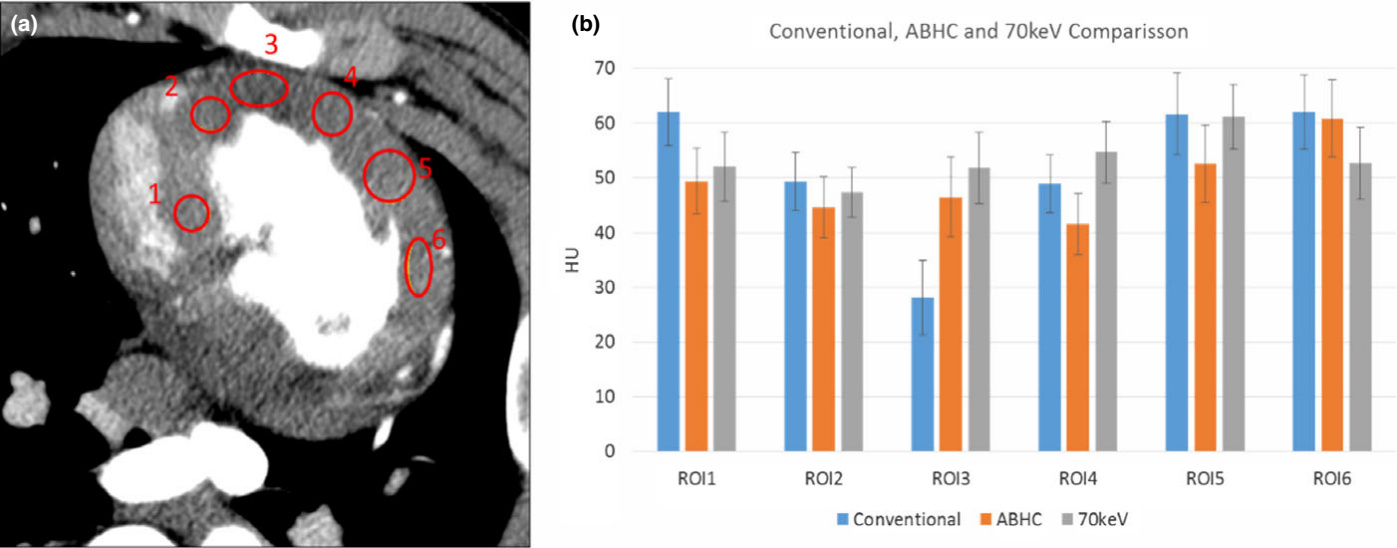
Comparison of mean HU values between (a) Conventional, (b) ABHC and 70 keV images in a baseline (FFR = 1.0) porcine model. Standard deviations of mean HU across ROIs are 13.26, 6.86, and 4.54 respectively. ROIs are shown in panel (a). At baseline, enhancement in the myocardium should be uniform. Reduction in HU in ROI3 is due to BH artifact and reduced by ABHC. W = 180/L = 50. [Color figure can be viewed at wileyonlinelibrary.com]

We also evaluated ABHC on *in vivo* images from our porcine model without coronary obstruction (FFR = 1), (Fig. 8). The graph compares standard CT with FBP, ABHC, and 70 keV images. Although we expect homogeneous perfusion in the myocardium in this nearly short axis view, there is some variation in the 70 keV image. Large variations in standard CT are very much reduced with ABHC. Standard deviations of mean HU across ROIs were 13.26, 6.86, and 4.54, respectively. BH artifact in ROI 3, the most affected and one that can be misconstrued as an LAD defect, was reduced from −23.7 to 5.2 HU.

We also compared BH correction capabilities with partial FOV. We found that as long as the structures that cause the majority of BH artifacts are present in the image, ABHC will be able to correct the image. Namely, for MPI‐CT, the ventricles, aorta, and bones close to the myocardium must be in the affected image for a successful BH correction. Highly attenuating structures, like ribs, that are further away from the myocardium, cause a BH artifact of around 1 HU. Their absence in the affected image will not allow the BH correction algorithm to use them for correction but the residual BH artifact should not exceed 1–2 HU.

**Figure 9:**
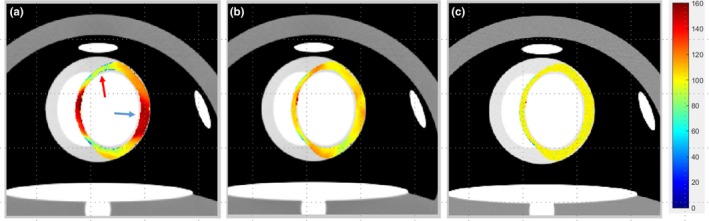
Comparison of absolute blood flow calculated from (a) conventional, (b) ABHC, and (c) 70 keV simulated perfusion scans. Simulated data have constant flow of 100 ml/min/100 g. Conventional had a less homogeneous blood flow compared to ABHC and 70 keV with coefficient of variations of 22%, 9%, and 5% respectively. In conventional images, BH artifacts causes false hypo‐perfusion (red arrow) and false hyper‐perfusion (blue arrow) which is corrected by ABHC. [Color figure can be viewed at wileyonlinelibrary.com]

Blood flow maps generated from simulated MPI‐CT, with homogeneous blood flow of 100 ml/min/100 g, using standard, ABHC, and 70 keV images are shown in Fig. 9. Image SNR in the myocardium at the peak enhanced image was ~9.8. Standard CT gives very high flows (blue arrow) as well as low flows (red arrow) with coefficient of variation of 22%. If one considers relative flows, these results would lead one to conclude the presence of a significant flow deficit. Flow is much more homogeneous in 70 keV and ABHC images as compared to standard CT. ABHC's 9% variation is comparable to physiologic variation of 70 keV at 5%.

**Figure 10:**
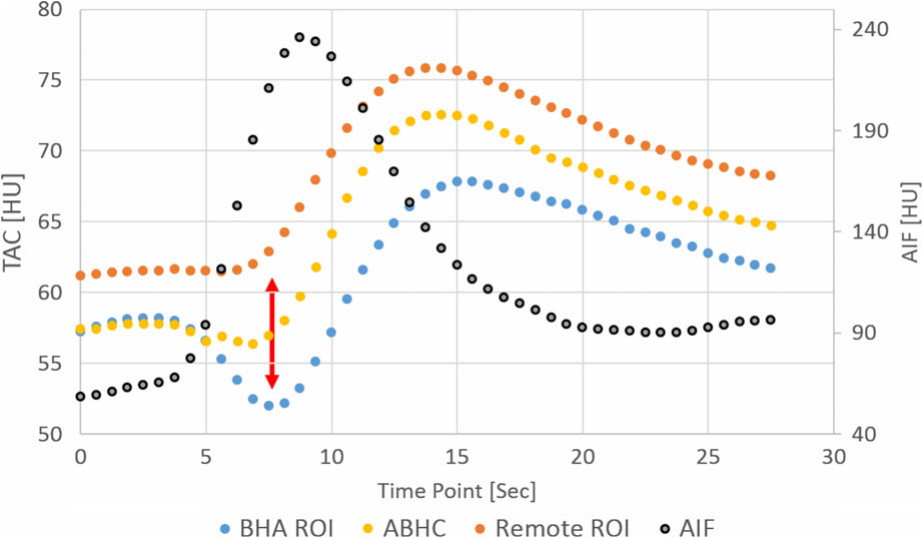
Effect of iodine‐filled LV on TACs. There is an observed depression (red arrow) in the TAC of a BH affected ROI near 11 o'clock [Fig. 9(a)] when the LV is filled with iodine. This will manifest as low blood flow in this ROI. ABHC reduces BH artifact in all affected time points, correcting this depression. For comparison, in a remote ROI at 1 o'clock, no depression is observed. The overall difference between affected ROI to remote ROI (~3 HU) is from BH artifact originated from the bones, regardless of iodine content. Note that the AIF taken from the ventricle was scaled down by 4 for presentation purposes. [Color figure can be viewed at wileyonlinelibrary.com]

The reason for the apparent flow deficit in the conventional image can be seen in TACs from an ROI near 11 o'clock of Fig. 9(a) (Fig. 10). Standard CT curves show a depression near the peak of the AIF due to BH which alters model fits as compared to the cases with ABHC and 70 keV. The remote ROI was chosen near 1 o'clock.

**Figure 11:**
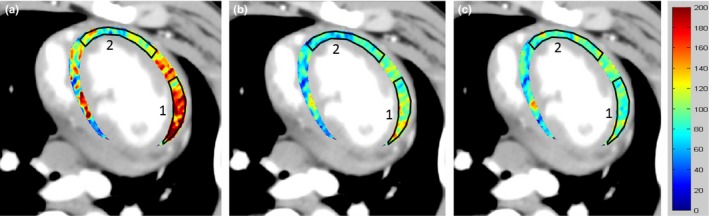
Comparison of absolute blood flow in porcine (FFR = 1.0) calculated from conventional (a), ABHC (b) and 70 keV (c) perfusion scans. Blood flow was calculated using the Johnson‐Wilson model. Conventional shows less homogeneous blood flow compared to ABHC and 70 keV, with flow ratios of 0.59, 0.85, and 0.93 respectively. Flow ratios were calculated as the ratio between the mean flow in ROI1 to the mean flow in ROI2. [Color figure can be viewed at wileyonlinelibrary.com]

Blood flow estimates with and without BH correction are compared for the porcine model with FFR = 1.0 in Fig. 11 to standard CT. Since it is common to report relative flows, blood flow ratios are given in the figure legend.

**Figure 12:**
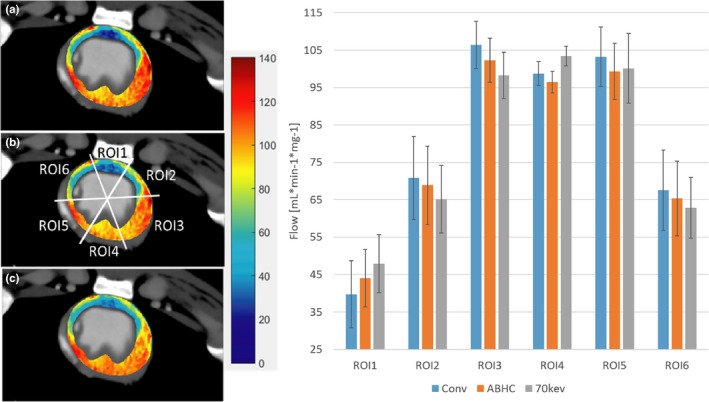
Comparison of absolute blood flow in porcine model at FFR = 0.7, calculated from conventional (a), ABHC (b) and 70 keV (c) images. BH artifact was effectively reduced without obscuring the flow deficit in the ischemic region. Blood flow calculated from ABHC images is closer to 70 keV than conventional, as the sum of the squared difference was reduced by 46%. Flow ratios for R0I‐6/ROI‐1 are 1.7, 1.48, and 1.31, for conventional, ABHC, and 70 keV image data, respectively. Data obtained from pig 17.

In Fig. 12, we measured FFR = 0.7 with an FFR wire following partial balloon occlusion in the LAD, expected to give a flow deficit in the LAD territory at about 9–3 o'clock. An appropriate flow deficit is shown in all three cases (standard CT, ABHC, and 70 keV). The myocardium was separated into sectors and results averaged (graph on right). Flow values with ABHC were much closer to the 70 keV reference than standard CT scans. After ABHC, a clear transmural perfusion gradient is evident which was previously obscured by BH artifacts. Importantly, ABHC did not “remove” the flow deficit and therefore did not create a false negative.

We investigated ABHC correction parameters on phantoms as a function of kVp and scanner. The ABHC algorithm robustly accounted for imaging at different kVp values (Table 1). We scanned the low‐concentration phantom four times at both 120 and 140 kVp on the SDCT scanner. Each scan contained 16 slices for a total of 64 individual slices (n = 64) at a single kVp. The high‐concentration phantom was scanned four times at 120 kVp (n = 64) and once at 140 kVp (n = 16). All scans were done at 100 mAs. Variation from one scan to the next (same scan protocol) as estimated by SD was minimal, with coefficient of variation typically about −0.22 and insignificant *P*‐value (*P* > 0.01). Comparing 120 and 140 kVP, we observed a statistically significant difference (*P* < 0.01) in both “a” and “b” parameters with the low‐iodine concentration phantom (Table 1). With the high‐concentration phantom, parameter “b” was significantly different but “a” showed an insignificant trend (*P* = 0.14). Comparing low‐ to high‐concentration phantom both scanned at 120 kVp or 140 kVp, also showed significant difference in both “a” and “b.” Mean and standard error (SE) can be seen in Table 1.

**Table 1:** Comparison between polynomial correction parameters “a” and “b” for low‐ and high‐concentration phantom scanned at 120 and 140 kVp. The SE for “b” translates into less than 1 HU value in the corrected image (average in a small ROI at 3 o'clock on Fig. 6b)

		“a” parameter		“b” parameter	
		120 kVp	140 kVp	120 kVp	140 kVp
Low‐concentration phantom	Mean	−0.12	−0.11	−1.63E‐06	−2.18E‐06
	SE	1.41E‐04	1.56E‐04	7.5E‐09	8.28E‐09
High‐concentration phantom	Mean	−0.04	−0.03	−2.3E−06	−2.74E‐06
	SE	1.56E‐04	5E‐04	1.4E‐09	6.25E‐09

We also compared correction parameters across scanners for *in vivo* imaging of pigs and patients. For each pig or patient, a specific slice was individually corrected for every time point with significant contrast agent along the MPI‐CT series. Comparing correction parameters for different pigs scanned with the same protocol on the same scanner, we found that values of “a” and “b” were not significantly different. However, when we compared the difference in correction parameters between scanners (all pigs scanned on iCT compared to all pigs scanned on SDCT compared to patients scanned on a different iCT), we found a significant difference in both “a” and “b” parameters (Table 2). Another way to examine the importance of subject‐specific parameter estimates is to compare results on flow maps. When we applied parameters from each of the pigs to the other pigs (all pigs scanned on the SDCT under the same protocol), we found a mean variation in flow of 1.9 100 ml/min/100 g, with standard deviation of 6.7 100 ml/min/100 g, and absolute maximum flow difference of 13.26 100 ml/min/100 g. It is worth noting that all pigs were of approximately the same size.

**Figure 13:**
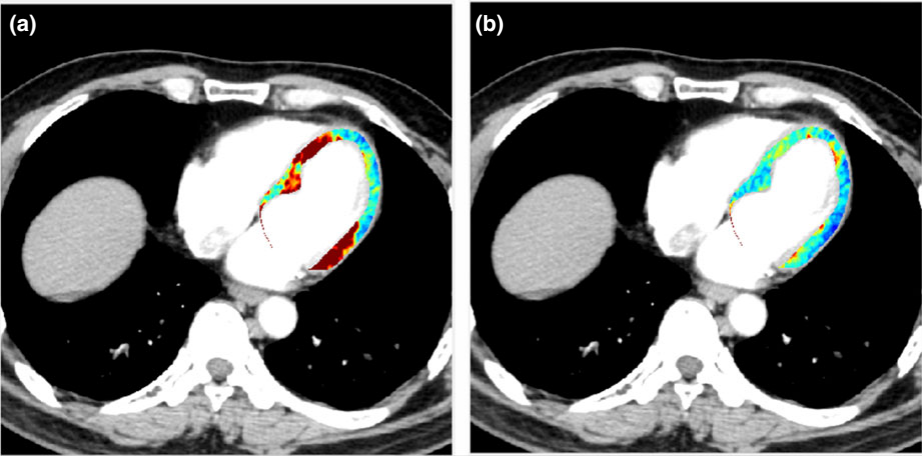
Blood flow estimation in clinical MPI‐CT, calculated from conventional (a) and ABHC (b) images. Blood flow calculated from ABHC images is more homogeneous. [Color figure can be viewed at wileyonlinelibrary.com]

Figure 13 shows blood flow estimation in clinical MPI‐CT. Blood flow calculated from ABHC images is more homogeneous compared to conventional.

**Table 2:** Comparison of correction parameters “a” (left) and “b” (right) between pigs scanned on iCT (N = 2) and SDCT (N = 11) and humans scanned on iCT (clinical, N = 2). *P*‐values for the “a” parameter were 3.53E‐03, 1.36E‐04, and 0.024 for iCT‐SDCT, SDCT‐Clinical, and iCT‐Clinical respectively. *P*‐values for the “b” parameter were 8.22E‐03, 5.37E‐04, and 5.04E‐03 for iCT‐SDCT, SDCT‐Clinical, and iCT‐Clinical respectively. All scans were done at 140kVp

	iCT	SDCT	Clinical
“a” parameter			
Mean	2.15E‐03	1.04E‐03	6.01E‐04
SE	9.43E‐06	2.16E‐06	7.59E‐06
“b” parameter			
Mean	−1.61E‐06	−1.74E‐06	−1.15E‐06
SE	3.12E‐09	1.36E‐09	2.19E‐08

**Figure 14:**
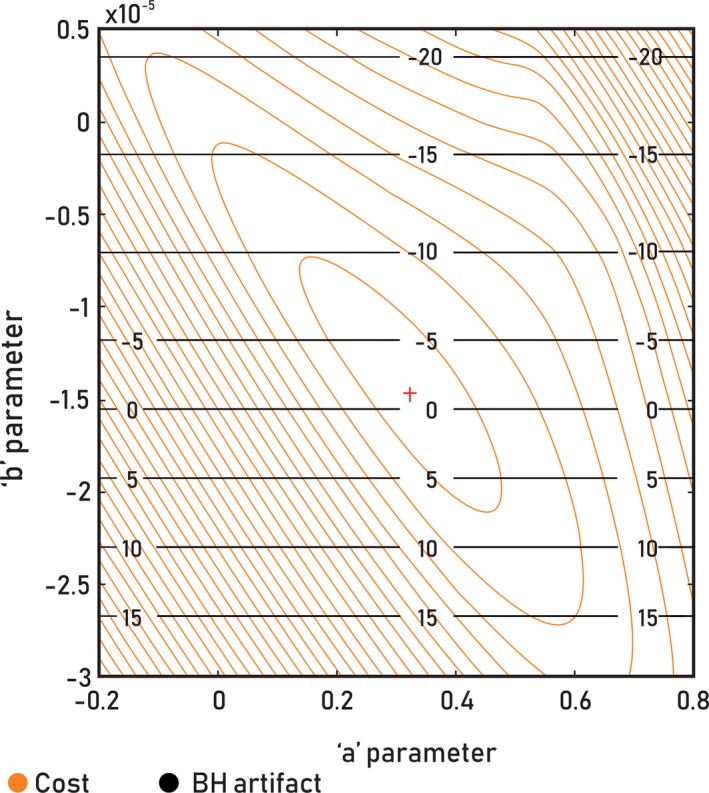
Example of the cost function sensitivity and BH artifact to the parameters “a” and “b.” The colored contours show the values calculated using the cost function. The black horizontal lines show the measured BH artifact. The red cross shows the minimum of the cost function as found by ABHC. Here, a change of ∼4 × 10^−6^ in “b” parameter causes a change of ~5 HU in BH artifact, while the “a” parameter does not affect BH artifact. [Color figure can be viewed at wileyonlinelibrary.com]

The sensitivity of the BH correction to the parameters “a” and “b” can be seen in Fig. 14. The colored contours show the value of the cost function for a range of “a” (*x*‐axis) and “b” (*y*‐axis) parameters. The black horizontal lines show the measured BH artifact. The red cross marks the minimum found by ABHC. According to this specific example (data obtained from pig 17), a change of ∼4 × 10^−6^ in “b” parameter causes a change of ~5 HU in BH artifact. As explained before, “a” parameter does not affect BH streaks artifact.

**Figure 15:**
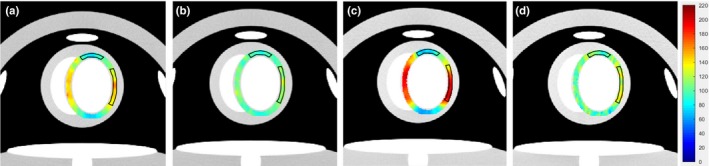
Comparison of simulated blood flow maps at different kVps. Simulated data have a constant flow of 100 ml/min/100 g. (a) and (c) were calculated from the conventional reconstruction of MPI‐CT images generated at 80 and 140 kVp respectively. (b) and (d) are the blood flow maps generated from ABHC corrected images of (a) and (c). ABHC found the appropriate correction parameters for each kVp. The flow ratio of the low flow region to the high flow region was increased from 0.61 to 0.85 at 80 kVp and from 0.43 to 0.89 at 140 kVp. In both cases, ABHC would change interpretation from a false positive for myocardial ischemia to a true negative. In both cases ABHC could have changed a clinical decision. Parameters (a, b) were (0.12, −1.79 × 10^−6^) and (0.14, −8.23 × 10^−6^), at 80 and 140 kVp, respectively. [Color figure can be viewed at wileyonlinelibrary.com]

A comparison of simulated blood flow maps obtained at different kVps is presented in Fig. 15. Simulated data have a homogeneous flow of 100 ml/min/100 g. Conventional reconstructed images (a) and (c) obtained at 80 and 140 kVp, respectively, show increased blood flow from 7 to 11 o'clock and from 2 to 5 o'clock of up to 211 ml/min/100 g and reduced blood flow as low as 73 ml/min/100 g in the rest of the myocardium. ABHC‐corrected images (b) and (d) show a more homogeneous blood flow. The flow ratio of the low flow region to the high flow region was increased from 0.61 to 0.85 at 80 kVp and from 0.43 to 0.89 at 140 kVp. A ratio greater than 0.80 is considered hemodynamically insignificant according to clinical FFR criteria.^44^ In both cases, ABHC would change the clinical interpretation from a false positive to true negative for myocardial ischemia. The correction parameters found by ABHC where a = 0.12, *b* = −1.79 × 10^−6^ for 80 kVp and a = 0.14, *b* = −8.23 × 10^−6^ for 140 kVp. BH artifact at 80 kVp compared to 140 kVp was 18.1 and 16.1 HU, respectively. Peak iodine contrast (peakEnhance), as computed by the difference between peak myocardial enhancement and the first time index, was much higher at 80 kVp than 140 kVp (i.e., 32.2 HU vs 16.3 HU, respectively). Taking the ratio of BHA/peakEnhance, we observe that there is a much reduced effect on the perfusion signal at 80 kVp (i.e., the ratios are 0.56 and 0.99, respectively).

## 4 Discussion

Reduction of BH artifacts is necessary for MPI‐CT, where artifacts are on the order of the myocardial iodine enhancement signal, leading to both qualitative hypo‐enhancement in individual CT images and inaccurate blood flow measurement in dynamic perfusion. The ABHC algorithm is an automatic, calibration‐free BH correction framework that can be applied to any scanner and imaging protocol. ABHC significantly reduces BH artifact in MPI‐CT, consequentially producing more accurate and reliable blood flow maps, demonstrated by reducing the false flow deficit at FFR = 1.0 and preserving ischemia at FFR <0.8 thus not creating false negatives. With the correction of false positives, MPI‐CT will improve its specificity, reducing the number of unnecessary referrals to invasive angiography. With accurate MPI‐CT, CT could become a preferred modality for noninvasive detection and rule‐out of coronary artery disease prior to invasive coronary angiography.

Previous work^45^ used TV as a cost function to achieve automatic BH correction. We found that adding the flatness term to the cost function produced a more robust and more accurate BH correction and blood flow estimation in the case of myocardial perfusion. The flatness term specifically targets the cupping phenomenon that is usually apparent in the LV.

In case of ischemia, the concern is that the algorithm can over‐correct the image and essentially “remove” the ischemia, creating a false negative. In this case, the TV part, computed over the myocardium, tries to give a constant value in the myocardium, which might pull the solution toward a “false negative.” However, the flatness term is computed over the LV and attempts to reduce cupping. It is sensitive to BH, but without consideration of the myocardium. Hence, we think it guards against an algorithm‐induced, false‐negative. Moreover, the extent of ischemia, whether it is LAD or RCA territories, is bigger than the BH artifact in most cases. For example, ischemia in LAD territory usually affects the antero‐septal and anterior regions of myocardium from about 9 o'clock to about 2–3 o'clock where most of the extent of BH is seen to be in the anterior region (between 11 o'clock and 12–1 o'clock) (Fig. 12). Since the correction algorithm is built on physical principles, and can only correct BH streaks, it cannot remove the whole ischemic region.

Various algorithms to assess cupping (e.g., TV, maximum difference between the highest and lowest values, and standard deviation) were investigated, but the one we used [Eq. (11)] gave the most robust results. It also tends to bring the signal within the ventricle to the appropriate value, an important result for proper flow estimation. Estimating flatness in the LV assumes a homogeneous iodine and blood mixture. To address this issue, we find ABHC's correction parameters using the three time points around peak enhancement. By these times, the LV is reasonably well mixed. For example, at peak enhancement, the coefficient of HU variation within the middle of the ventricle is ~5% as compared to 35–40% at early time points. We used the ventricle because the aorta was obscured by artifact from the FFR wire. In a clinical setting, we believe it will be advantageous to assess cupping and measure the AIF in the aorta instead of the LV. The aorta will have a better mixture of blood and iodine and it does not have internal structures like trabeculae. Furthermore, it is easier to automatically segment due to its round shape. We found that the cupping term improves the accuracy of quantitative HU values, as determined from digital and physical phantoms (e.g., see Fig. 6), although accurate restoration of quantitative HU values might be challenging without calibration. Furthermore, we found that adding the filtration step before evaluating the cost of an image, greatly improved robustness and accuracy of the correction algorithm yielding reproducible results across a range of object sizes, noise levels, FOV and different scan protocols. The optimization problem is nonconvex. Before adding the filtration step, the cost function had multiple local minima. The main cause was that most of the signal of TV came from noise, a high‐frequency phenomenon, compared to BH which is a low‐frequency phenomenon. However, after applying the proposed filtration, the local minima are removed and the objective function appears to smoothly approach a global minimum.

The use of BH correction algorithms enables MPI‐CT on conventional single‐energy CT scanners. While energy‐sensitive CT has the ability to significantly reduce BH artifacts, many sites do not have access to these scanners. Energy‐sensitive CT systems include kV switching, dual source, dual layer detector, and multi‐spectral technologies. Typically, there is a dose penalty for energy‐sensitive CT, including dual source imaging,^46^ which may give a clinical preference to conventional CT for MPI‐CT. With conventional CT, recent developments in model‐based image reconstruction enable low‐dose imaging. Such technologies could bring x‐ray dose down to levels similar to or below that of cardiac SPECT imaging.^47^

We analyzed the need for subject, scanner, and kVp specific BH correction. Comparing the correction parameters that ABHC found for different pigs on one specific scanner with the same scanning protocol, we observed that there is no significant difference. We attribute that to the fact that the correction parameters should be about the same for a specific scanner using a specific protocol regardless to the subject being scanned, especially if the subjects are close in build/weight. However, when we compare different protocols or different scanners, we found a measurable difference in the correction parameters. We observed a much more accurate blood flow maps after using a scanner specific, protocol specific correction parameters compared to using the same parameters for different scans. Comparing the low‐concentration phantom to the high‐concentration phantom scanned with the same protocol gave us significant differences in both “a” and “b.” This supports findings in other similar experiments^48^. We believe that a second degree polynomial is a good approximation to the correction, but it is not ideal, especially when a large difference in iodine concentrations are involved. We deliberately used higher than clinical concentrations in the high‐concentration phantom. This also explains why we did not observe the same behavior between different pigs or different humans scanned with the same protocol. In those cases, about the same iodine concentration was used. A higher degree polynomial, or dividing the line integrals values (projections) into several ranges, corresponding to varied iodine concentrations, and finding a different polynomial for each range^48^ could produce more accurate results. When correcting a set of MPI‐CT images, we found it desirable to use a single set of correction parameters. When each image is corrected using different correction parameters, we observed fluctuations in TACs giving inconsistent blood flow estimation.

Our experiments have drawn attention to an unexpected effect of kVp on perfusion flow estimates. Although many would likely argue that high kVp would be better than low kVp for limiting the effect of BH on perfusion flow estimation, this is not the case (see Fig. 15). Indeed, absolute BH artifacts are more pronounced at low kVp than at high kVp. However, since the iodine signal in the myocardium is much higher at low relative to high kVp, the relative size of BH to perfusion signal is less at 80 kVp than 140 kVp. After using ABHC, this phenomenon is eliminated and we get good flow estimation at both kVp values.

ABHC should enable MPI‐CT with any existing conventional scanner suitable for dynamic cardiac acquisition. Although some vendors provide an iodine BH correction method for their specific conventional scanners, ABHC could provide a means of harmonizing results across all scanners. The addition of accurate MPI‐CT to coronary CT angiography could make CT a preferred modality for noninvasively determining significant obstructive coronary artery disease and potential microvascular disease prior to invasive coronary angiography.
